# Congenital Central Hypoventilation Syndrome: What to Expect During Pregnancy

**DOI:** 10.7759/cureus.17827

**Published:** 2021-09-08

**Authors:** Abdelrahman Yousif, Ajah Chandler, Malek Ghandour, Atinuke Akinpeloye

**Affiliations:** 1 Obstetrics and Gynecology, Michigan State University, Flint, USA; 2 Obstetrics and Gynecology, Wayne State University, Detroit, USA

**Keywords:** fetal complications, fetal growth restriction, prenatal care, feto-maternal outcome, prenatal genetic testing, central congenital hypoventilation

## Abstract

Congenital central hypoventilation syndrome (CCHS) is a rare autosomal dominant disease that affects the autonomic regulation of breathing. Patients present with respiratory symptoms such as sleep apnea and dependency on mechanical ventilation during sleep or nonrespiratory symptoms such as orthostatic hypotension and sinus bradycardia. CCHS in the neonates are linked but not limited to Hirschsprung disease, neural crest cell tumors, and esophageal dysmotility. Literature about CCHS management in pregnancy is scarce. Several studies have shown that pregnant CCHS patients are at risk of adverse pregnancy outcomes such as preterm delivery, low birth weight, and maternal complications including increased dependency on the mechanical ventilation and sometimes cardiopulmonary arrest. A multidisciplinary approach has been shown to be associated with better pregnancy outcomes. In this case report, we present a case of a patient with CCHS who had her prenatal care at our high-risk pregnancy unit and delivered a healthy baby. We encourage having a thorough discussion with such high-risk patients throughout their prenatal care or even preconception about their pregnancy expectations and outcomes in order to provide them and their babies with the care needed in the postpartum period.

## Introduction

Congenital central hypoventilation syndrome (CCHS) results from autonomic dysfunction and alveolar hypoventilation secondary to paired-like homeobox 2B (PHOX2B) gene mutations [1-2]. Current literature describes the PHOX2B gene as a vital gene in the human embryonic autonomic nervous system (ANS) developmental process [1-2]. Repeated polyalanine expansions in the PHOX2B gene are linked to symptoms’ development and severity [1-2]. A patient could present with either respiratory or nonrespiratory symptoms such as sleep apnea, orthostatic hypotension, and sinus bradycardia [1-4]. 

The prevalence of CCHS is 1:200,000 births [5]. Diagnosis of CCHS requires PHOX2B genetic testing once suspected based on family history and symptoms profile. PHOX2B gene mutations lead to other neurocristopathies, such as Hirschsprung disease and esophageal dysmotility, which can also be initial presentations of the syndrome [1,2,6]. While most affected individuals present in the neonatal period, pregnant women with a diagnosis of CCHS are vulnerable to maternal and fetal complications. Respiratory symptoms tend to deteriorate during pregnancy up to cardiopulmonary arrest. Some patients require mechanical ventilation with positive pressure ventilation (PPV), noninvasive positive pressure ventilation (NPPV), or diaphragmatic pacing (DP) [2,4]. Reports have linked CCHS with chronic uteroplacental insufficiency with subsequent increased risk of preterm delivery, gestational hypertension, intrauterine growth restriction, preeclampsia, and unplanned cesarean section [7-8].

This case report will discuss a pregnant woman with a diagnosis of CCHS who had an uneventful pregnancy with good perinatal outcomes after receiving her prenatal care at our high-risk unit. The literature has been scarce about congenital central hypoventilation syndrome in pregnancy. In few case reports, respiratory adjustments in form of mechanical ventilation settings change are made throughout the pregnancy to cope with the physiological changes in pregnancy; however, in our patient, no changes were needed, and patient course was stable throughout her pregnancy. 

## Case presentation

A 23-year-old patient gravida (G) 1 para (P) 0 presented at 20 weeks of gestational age (GA) for initial prenatal care. The patient has a medical history of asthma and congenital central hypoventilation syndrome genotype PHOX2B 20/25. At the age of one month old, a tracheostomy was performed on this patient. Since then she has been ventilator dependent when sleeping. The ventilator settings are tidal volume (VT) 550 ml, respiratory rate 20 breaths per minute, inspiratory time 1.5 seconds, positive end-expiratory pressure (PEEP) 0 mmhg, and model Newport Ht70 mode assist control (AC). The patient initiated her prenatal care at the maternal-fetal medicine unit at 20 weeks of gestational age. At 24 weeks of gestation, her anatomy scan showed normal fetal anatomy. Routine second trimester labs' results including hemoglobin and hematocrit, 75 mg glucose tolerance test, HIV antibodies, and syphilis antibodies were within normal limits. Her growth ultrasound at 33 weeks showed an appropriate fetal growth for the gestational age, estimated fetal weight of 1,975 gm (4 lb 6 oz). 

A multidisciplinary team consisting of maternal-fetal medicine specialists, pulmonologists, and neonatologists followed the patient throughout the pregnancy. At 39 weeks and 3 days, the patient presented to the triage for contractions. On cervical exam, the patient was 3 cm dilated, 80% effaced, and -2 for fetal station. Labor augmentation followed using oxytocin drip. Under continuous intrapartum fetal monitoring, the patient’s labor course was uneventful. Within hours of labor augmentation, spontaneous rupture of membranes occurred, followed shortly after by the onset of the second stage of labor. Less than an hour afterward, the patient had an uncomplicated vaginal delivery within an hour from complete cervical dilation. The newborn Appearance, Pulse, Grimace, Activity, and Respiration (APGAR) scores at 1 and 5 minutes were 4 and 8, respectively. In the postpartum period, the patient recovered well and had a negative postpartum depression screening questionnaire with appropriate family support. 

## Discussion

CCHS is a rare but highly concerning genetic disorder that can pose significant complications in pregnant patients and their newborns. The autonomic dysfunction in CCHS is associated with alveolar hypoventilation that can lead to deleterious effects including decreased respiratory drive, apnea, sinus bradycardia, sinus pause, and orthostatic hypotension [1,2]. 

Respiratory physiological changes occur in pregnancy including hypercapnia, decrease in the minute ventilation, and the pressure exerted by the gravid uterus restricting the movement of the diaphragm [3,9]. Meanwhile, patients with CCHS start with a lower minute ventilation at baseline secondary to a lack of central respiratory drive which makes CCHS pregnant patients at higher risk of acute hypoxia and acidosis in pregnancy [9]. Herein, it is imperative to monitor oxygenation during pregnancy and adjust the ventilator settings as needed [1,3,10]. On the other hand, fetal complications such as growth retardation have also been diagnosed in pregnant CCHS patients [10]. Monitoring for fetal complications is important as the states of hypoxemia and hypercarbia impact the fetus [10].

A meta-analysis on obstetrics adverse outcomes associated with sleep-disordered breathing in pregnant women showed an increase in preterm births and low birth weight cases [11]. CCHS is an inherited autosomal dominant disease with variable penetrance and expressivity. Multiple individuals of a family may have the genotype; a wide range of physical symptoms can inconsistently be present among people with the same genotype. When a family member is diagnosed with CCHS, monitoring and genetic testing is recommended for the neonates and the rest of the family members [4]. Patients with CCHS present with a wide spectrum of symptoms including altered basal body temperature, breath-holding spells, lack of perception of dyspnea, exercise intolerance, and lack of physiological response to different environmental stressors. It is recommended to perform genetic tests on the family pedigree that is vulnerable for inheriting the condition, especially infants born to affected mothers [3]. 

Most patients are diagnosed during the neonatal period; however, some patients present with CCHS during adulthood and, more specifically, during pregnancy [4,5,10]. The mutations in the PHOX2B gene have been theorized to correlate with a diagnosis of CCHS [1]. Repeat expansion mutations were found to be either polyalanine associated or nonpolyalanine associated in the PHOX2B gene. One study stated that increased polyalanine repeat expansions were associated with increased ventilator dependency in patients [2,12]. 

## Conclusions

In pregnant patients with CCHS, prenatal care and follow-up is of utmost importance. Detailed prenatal counseling will provide patients with information to make informed decisions about their care and their offspring. Discussion with a multidisciplinary team including an obstetrician, hematologist, neurologist, pulmonologist, psychologist, and physiotherapist, about their options from elective abortion to the place of delivery, will ensure that the affected neonates receive the needed care to transition to extrauterine life and optimize the pregnancy outcomes. Further research into the implications of cardiorespiratory disorders such as CCHS in pregnancy is crucial to understand both the disease mechanism and diagnosis and will lead to better maternal and fetal outcomes during pregnancy.
